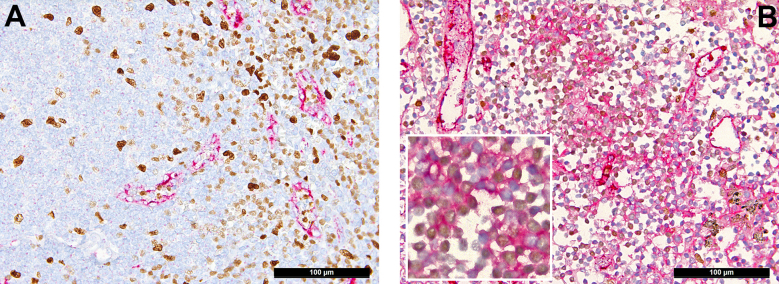# Von Willebrand factor and the thrombophilia of severe COVID-19: *in situ* evidence from autopsies

**DOI:** 10.1016/j.rpth.2023.100182

**Published:** 2023-05-18

**Authors:** Jana van den Berg, Jasmin D. Haslbauer, Anna K. Stalder, Anna Romanens, Kirsten D. Mertz, Jan-Dirk Studt, Martin Siegemund, Andreas Buser, Andreas Holbro, Alexandar Tzankov

**Affiliations:** 1 Department of Hematology, University Hospital Basel, Basel, Switzerland; 2 Department of Pathology, Institute of Medical Genetics and Pathology, University Hospital Basel, Basel, Switzerland; 3 Department of Pathology, Cantonal Hospital Baselland, Liestal, Switzerland; 4 Department of Oncology, Cantonal Hospital Baselland, Liestal, Switzerland; 5 Department of Medical Oncology and Hematology, University Hospital Zürich, Zürich, Switzerland; 6 Intensive Care Unit, Department of Acute Medicine, University Hospital, Basel, Switzerland; 7 Regional Blood Transfusion Service, Swiss Red Cross, Basel, Switzerland

**Keywords:** ADAMTS13 protein, COVID-19, immunohistochemistry, SARS-CoV-2, thrombosis, von Willebrand factor

## Abstract

**Background:**

COVID-19 is accompanied by a hypercoagulable state and characterized by microvascular and macrovascular thrombotic complications. In plasma samples from patients with COVID-19, von Willebrand factor (VWF) levels are highly elevated and predictive of adverse outcomes, especially mortality. Yet, VWF is usually not included in routine coagulation analyses, and histologic evidence of its involvement in thrombus formation is lacking.

**Objectives:**

To determine whether VWF, an acute-phase protein, is a bystander, ie, a biomarker of endothelial dysfunction, or a causal factor in the pathogenesis of COVID-19.

**Methods:**

We compared autopsy samples from 28 patients with lethal COVID-19 to those from matched controls and systematically assessed for VWF and platelets by immunohistochemistry. The control group comprised 24 lungs, 23 lymph nodes, and 9 hearts and did not differ significantly from the COVID-19 group in age, sex, body mass index (BMI), blood group, or anticoagulant use.

**Results:**

In lungs, assessed for platelets by immunohistochemistry for CD42b, microthrombi were more frequent in patients with COVID-19 (10/28 [36%] vs 2/24 [8%]; *P* = .02). A completely normal pattern of VWF was rare in both groups. Accentuated endothelial staining was found in controls, while VWF-rich thrombi were only found in patients with COVID-19 (11/28 [39%] vs 0/24 [0%], respectively; *P* < .01), as were NETosis thrombi enriched with VWF (7/28 [25%] vs 0/24 [0%], respectively; *P* < .01). Forty-six percent of the patients with COVID-19 had VWF-rich thrombi, NETosis thrombi, or both. Trends were also seen in pulmonary draining lymph nodes (7/20 [35%] vs 4/24 [17%]; *P* = .147), where the overall presence of VWF was very high.

**Conclusion:**

We provide *in situ* evidence of VWF-rich thrombi, likely attributable to COVID-19, and suggest that VWF may be a therapeutic target in severe COVID-19.

## Essentials

• In COVID-19, von Willebrand factor (VWF) is highly elevated and predictive of adverse outcomes.
• It is unclear whether VWF only marks endothelial damage or promotes COVID-19–associated coagulopathy.
• When autopsy samples were compared with matched controls, VWF-rich thrombi were more frequent in samples from patients with COVID-19.
• VWF seems to contribute to thrombosis in COVID-19 and may be a therapeutic target.

## 1 Introduction

COVID-19, caused by SARS-CoV-2, was declared a global pandemic in March 2020. Despite the development of effective vaccines and progress in understanding of its pathomechanism and some treatment options [1], COVID-19 still continues to challenge health care systems across the globe. While the majority of patients experience only mild symptoms, such as cough and fever, COVID-19 can also cause progressive organ and, particularly, respiratory failure [[2], [3], [4]]. Furthermore, COVID-19 is accompanied by a hypercoagulable state [5], characterized by microvascular and macrovascular thrombotic complications, which contribute substantially to morbidity and mortality [[5], [6], [7]]. For patients with severe disease in need of intensive care unit (ICU) hospitalization—including more recent COVID-19 variants of the omicron strain, which are considered generally less severe, with infrequent hospitalizations and ICU admissions—mortality is high (∼30% in the ICU, including our center [3,[8], [9], [10], [11]]) and therapeutic options are still imperfect [1,12]. Furthermore, survivors—even after mild disease—can experience persistent symptoms, known as post-COVID syndrome (long COVID) [13], which may also be related to the hypercoagulable state [14,15]. In line with these observations, postmortem findings reveal diffuse alveolar damage (DAD) and endothelial dysfunction, eg, accompanied by microthrombosis of pulmonary vasculature. Thrombotic complications were shown to be significantly more prevalent in patients with COVID-19 than in those with DAD due to other causes [[16], [17], [18], [19], [20]].

Initial reports on COVID-19 suggested a dysregulation of hemostasis, reflected by elevation of D-dimer [2,7], which are significantly increased, especially in critically ill patients and nonsurvivors [21,22], and correlate with a poor outcome [[22], [23], [24], [25]]. Initially described as elevated among other markers of endotheliopathy and glycocalyx damage [[26], [27], [28]], von Willebrand factor (VWF) seems to even more accurately predict outcomes, especially mortality, in patients with COVID-19 [21,22,[29], [30], [31], [32], [33]]. Physiologically, VWF is mainly synthesized in endothelial cells [34], and its multimers are either constitutively released from the endothelium or stored within endothelial granules in the form of ultralarge VWF multimers [35]. In the bloodstream, these are cleaved by the plasmatic enzyme disintegrin-like and metalloprotease with thrombospondin repeats 13 (ADAMTS13). Upon tissue damage, shear stress, or inflammatory cytokine elevation (eg, tumor necrosis factor-α, interleukin [IL]-6, or IL-8), VWF is rapidly released into the bloodstream [35,36]. In addition to interacting with platelets, especially in thrombus formation, VWF has further relevant functions: it is a carrier of coagulation factor VIII and actively contributes to inflammatory processes by binding to neutrophil extracellular traps, recruiting leukocytes to sites of vascular damage or inflammation [[36], [37], [38]]. Recently, VWF was also shown to be involved in angiogenesis [[39], [40], [41]]. An imbalance between VWF and ADAMTS13 is pathognomonic for thrombotic thrombocytopenic purpura, a thrombotic microangiopathy, in which uncleaved VWF, due to severe deficiency in the function of ADAMTS13, causes microthrombosis, thrombocytopenia, and microangiopathic hemolytic anemia, eventually leading to organ ischemia. While hemolytic anemia does not seem to be present and thrombocytopenia is usually mild [42,43] in patients with severe COVID-19, imbalance in the ADAMTS13-VWF axis has been repeatedly documented [22,31,44,45]. Plasma VWF levels are immensely elevated and hyperactive, while ADAMTS13 activity is low, which is predictive of poor outcome [6,21,[29], [30], [31], [32],^46^].

Given the severe endothelial damage and hyperinflammation in patients with COVID-19 and the physiologic functions of VWF, it is conceivable that VWF plays a role in thrombosis in COVID-19. However, as VWF is also an acute-phase protein [37], whether it is a bystander or a relevant factor in the pathogenesis of COVID-19 should be carefully evaluated. While several studies were conducted on plasma levels, histologic evidence is currently scarce, limited to a few autopsy studies [20,47].

In this study, we evaluated the *in situ* distribution of VWF in human tissues (lungs, pulmonary draining lymph nodes, and hearts) in a COVID-19 autopsy cohort in comparison with that in matched controls with analogous sequelae.

## 2 Methods

### 2.1 Patient cohort and study design

Twenty-eight cases of lethal COVID-19 (confirmed via nasal swab before death using polymerase chain reaction [PCR]; all tissues were real-time reverse-transcription (RT)-PCR controlled and quantified for SARS-CoV-2), 24 carefully selected lung control tissues, 9 heart control tissues, and 23 lymphoid control tissues were enrolled in this study. Autopsies of patients with lethal COVID-19 were performed as previously reported [18,48,49]. Patients with COVID-19 died between March and May 2020. The virus was of the B.1 lineage without mutations that later defined variants of concern (eg, alpha, beta, delta, or omicron), as deduced from whole-genome sequencing of a subset of samples (n = 9) and epidemiologic data [50].

Controls were selected from archived tissues from the Institute of Pathology at the University Hospital of Basel and chosen to match as best as possible the COVID-19 cohort with respect to age, sex, body mass index (BMI), and sequelae. The database was searched by cause of death and sequelae, and patients were included if the causative agent was apparent (ie, respiratory failure or DAD caused by influenza). All control tissues from lungs and hearts stemmed from autopsies [48,51]. Lung tissue donors had died of respiratory failure (n = 19) and cardiac death (n = 5). They included histomorphologically unremarkable lungs from the latter, and pneumococcus pneumonia, influenza pneumonia, and non–COVID-19 DAD were caused either by infections by other pathogens (*Enterococcus faecalis*, *Candida albicans*, *Klebsiella pneumonia*, and *Pseudomonas spp.*) or toxicity (drug-induced, post–stem cell transplant, or postoperative lung injury). Heart tissue samples were derived from patients without COVID-19 but with similar sequelae (non–COVID-19 DAD, pulmonary embolisms, and bronchopneumonia). Lymph node controls comprised 5 autopsy samples of patients who died of non–COVID-19 respiratory failure (pneumonia and non–COVID-19 DAD; same patients from whom the lung tissue samples were included) and 18 samples from lymph node biopsies from patients with similar sequelae (infectious mononucleosis lymphadenopathy, non–SARS-related hemophagocytic lymphohistiocytosis, mucosa-associated lymph nodes with extrafollicular plasmablast activation, follicular hyperplasia, and unremarkable mediastinal lymph nodes mainly from tumor staging [49]). Clinical data were extracted from electronic health records. Ethnicity was not routinely collected in the control group, yet by experience, consisted of >95% White patients. The COVID-19 cohort comprised 1 Black and 27 White patients.

### 2.2 Histologic assessment

All tissue samples were processed with standard (ISO15189-accredited) histochemical procedures and stained with hematoxylin and eosin. Tissue microarrays were constructed by selecting 3 areas of interest/tissue samples from the collected diagnostic probes as previously reported [49]. Immunohistochemistry was performed on the tissue microarrays using the automated staining system Benchmark XT (Roche/Ventana Medical Systems), except for fibrin staining, which was performed manually, as previously described [18]. The antibodies and protocols used are shown in Supplementary Table S1.

**Supplementary Figure 1:**
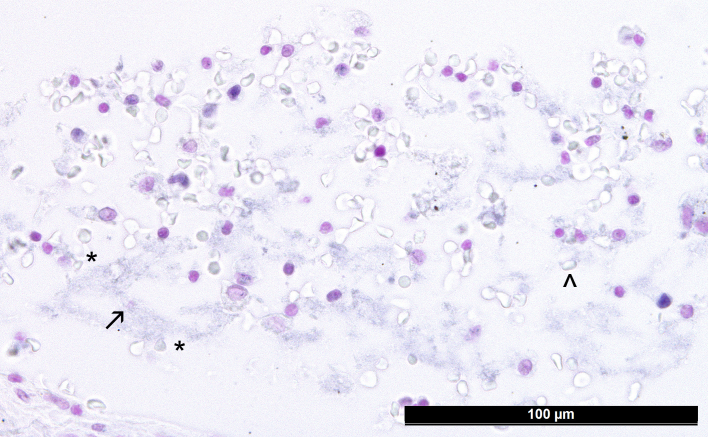


Histologic assessment was performed by board-certified pathologists (J.D.H. and A.T.) on a Leica DM4B microscope at a magnification of 200× or 400× using following criteria: CD42b staining of single platelets in entrapped blood of organs was considered normal; additionally, CD42b^+^ platelets in granulocyte-rich NETosis thrombi, CD42b^+^ platelets in compact fibrin-rich thrombi, CD42b^+^ platelets in hyaline membranes of DAD (lungs only), >10 platelets/0.19 mm^2^ of subcapsular sinus (lymph nodes), and presence of CD42b^+^ megakaryocytes were each scored as either present or absent; fibrin^+^ microthrombi were scored as either absent or present; and VWF staining confined to the endothelial lining of vessels or in a net-like/capillary pattern (lungs) and weak VWF staining in macrophages were considered normal, while presence of accentuated capillary VWF staining without thrombi and VWF thrombi in medium-sized and small vessels or sinus (lymph nodes), presence of VWF in granulocyte-rich NETosis thrombi and VWF-coated desquamated cells in DAD (lungs only), and presence of >5% VWF^+^ histiocytes in lymph nodes were each documented as either absent or present. NETosis was confirmed by immunohistochemistry for citrullinated histone H3 and DNA counterstain by the Feulgen method (Supplementary Figure S1) as previously described [52]. In brief, in a subgroup of 7 patients and 15 controls of this cohort, extensive neutrophil infiltration, with characteristic myeloperoxidase and citrullinated histone H3 staining patterns within thrombi associated with DNA stain, diagnostic of NETosis, was observed and linked to the obtained data for purpose of the present study.

### 2.3 Statistical analysis

Statistical analyses were performed with IBM SPSS, version 28. For categorical data, Fisher exact test was applied. For continuous nonparametric variables, Mann–Whitney U-test was used. Correlation analyses were calculated with Spearman ρ. Time from symptom onset to death (SOTD) survival was estimated applying the Kaplan-Meier method and log-rank test. *P* values of <.05 were considered significant.

## 3 Results

### 3.1 Patient and disease characteristics

A total of 28 COVID-19 cases were included in this study, with a male-to-female ratio of 20:8 (ie, 71% men) and a median age at diagnosis of 78 years (range, 53-96 years). The median hospitalization time before death was 6 days (range, 0-37 days). The median SOTD time was 14 days (range, 3-45 days). Ten patients were admitted to the ICU, where 8 received invasive mechanical ventilation. Two patients died in the emergency room on the day of admission. Eighty-three percent (20 of 24 patients for whom this information was available) of patients received anticoagulation during hospitalization (7/24 [29%] with therapeutic and 13/24 [54%] with prophylactic dosage), and 30% (6/20) had been treated with anticoagulation before admission (4/20 [20%] with therapeutic and 2/20 [10%] with prophylactic dosage). The use of anticoagulants was similar in the control cohort (Table 1). Prophylactic dosage mainly consisted of 50 to 100 IU/kg body weight of dalteparin administered subcutaneously once daily. Therapeutic anticoagulation included 200 IU/kg body weight of dalteparin (monitored based on anti-factor Xa activity), phenprocoumon (monitored based on international normalized ratio [INR]), or rivaroxaban/apixaban at therapeutic dosage. Further information on medication, comorbidities, blood group distribution, and laboratory findings are summarized in Supplementary Tables S2 and S3.

**Table 1:** Baseline and clinical characteristics of patients with COVID-19 vs those of controls.

	COVID-19	Control lungs	Control hearts	Control lymph nodes	All controls
	(*n* = 28)	(*n* = 24)	(*n* = 9)	(*n* = 23)	(*n* = 51^a^)
**Baseline**					
Age, y, median, (IQR)	78 (22)	76 (17)	67 (17)	57 (34)	66 (33)
Sex, male, n (%)	20 (71%)	14 (58%)	8 (89%)	13 (57%)	33 (65%)
BMI, kg/m^2^, median (IQR)	28 (10)	27 (9)	25.5 (8)	27 (4)	27 (8)
**Clinical data**					
Median hospitalization time, days (IQR) (*n* = data on)	6 range, 1-37; IQR, 11; n = 27	7 range, 3-50; IQR, 10; n = 8	14 range, 1-56; IQR, 26; n = 8	6 range, 3-50; IQR, 13; n = 6	7 range, 3-50; IQR, 10; n = 9
ICU admission (%)	10/26 (39%)	16/23 (70%)	5/8 (63%)	6/18 (33%)	22/44 (50%)
Mechanical ventilation (%)	8/27 (30%)	7/23 (30%)	3/8 (38%)	4/18 (22%)	11/44 (25%)
Days from symptom onset to death, median (IQR)	14 range, 3-45; IQR, 14; n = 23	n/a	n/a	n/a	n/a
Anticoagulation	20/24 (83%)	18/20 (90%)	5/7 (71%)	9/14 (64%)	27/36 (75%)
Therapeutic (%)	7/24 (29%)	8/20 (40%)	4/7 (57%)	2/14 (14%)	12/36 (33%)
Prophylactic (%)	13/24 (54%)	10/20 (50%)	1/7 (14%)	7/14 (50%)	15/36 (42%)

Baseline and clinical characteristics of patients with COVID-19 were not significantly different from those of controls, with the exception that the rate of admission to the intensive care unit was higher in the control lung group and age was lower in control lymph node donors.

n/a, not available.

a Partially overlapping patients (ie, lung and lymph node specimens from identical patients); therefore, n ≠ the sum of the n of specific organs.

**Table 2:** Results - Histologic findings in COVID-19 compared to control.

Lung	COVID-19	Controls	*P* value
DAD	28/28 (100%)	12/23 (52%)	<.001
Exudative	14/28 (50%)	5/12 (42%)	
Combined	9/28 (32%)	3/12 (25%)	
Proliferative	5/28 (18%)	4/12 (33%)	
qRT-PCR for SARS-CoV-2	Positive: 23/27 (85%)	n/a	
	Negative: 4/27 (15%)		
Lung, VWF^a^			
Normal pattern	2/28 (7%)	6/24 (25%)	.081
Desquamated cells in the alveoli	20/28 (71%)	16/24 (67%)	.471
Accentuated capillary staining^b^	3/28 (11%)	7/24 (29%)	.092
VWF-rich thrombi (all)	11/28 (39%)	0/24 (0%)	<.001
Small vessels	6/28 (21%)	0/24 (0%)	.019
Medium-sized vessels	7/28 (25%)	0/24 (0%)	.009
NETosis thrombi enriched with VWF	7/28 (25%)	0/24 (0%)	.009
VWF-rich thrombi or NETosis thrombi enriched with VWF^c^	13/28 (46%)	0/24 (0%)	<.001
Lung, platelets (CD42b)^d^			
Normal pattern	9/28 (31%)	15/24 (63%)	.028
Platelets within hyaline membranes	5/28 (17%)	0/24 (0%)	.038
Single scattered megakaryocytes	4/28 (14%)	2/24 (8%)	.674
Compact platelet-rich microthrombi	10/28 (36%)	2/24 (8%)	.02
NETosis thrombi enriched with platelets	17/28 (61%)	9/24 (38%)	.082
Lung, fibrin^e^			
Capillary microthrombi	12/28 (43%)	2/24 (8%)	.005

Heart, VWF	COVID-19	Control	*P* value
qRT-PCR for SARS-CoV-2	Positive: 9/20 (45%)	n/a	
	Negative: 11/20 (55%)		
Capillary microthrombi^a^	10/20 (50%)	4/9 (44%)	.55

Lymph nodes	COVID-19	Control	*P* value
qRT-PCR for SARS-CoV-2	Positive: 12/19 (63 %)	n/a	
	Negative 7/19 (37 %)		
Fibrin microthrombi^e^	6/19 (32%)	0/14 (0%)	.024
Lymph nodes, platelets^d^			
NETosis thrombi enriched with platelets	5/18 (28%)	4/23 (17%)	.336
Increased presence of platelets in sinus	10/18 (56%)	8/23 (35%)	.156
Lymph nodes, VWF^a^			
VWF thrombi	7/20 (35%)	4/24 (17%)	.147
More than 5% VWF^+^ histiocytes^a^	8/20 (40%)	3/14 (21%)	.038

DAD was assessed on hematoxylin and eosin stained slides.

DAD, diffuse alveolar damage; n/a, not available; qRT-PCR, quantitative real-time reverse-transcription-polymerase chain reaction; VWF, von Willebrand factor.

a Histologic findings in COVID-19 lung tissue, the myocardium, and pulmonary draining lymph nodes compared with those in respective controls utilizing staining for VWF (FVIII-R).

b Accentuated capillary staining without thrombi.

c In COVID-19, 46% of patients had VWF-rich thrombi, NETosis thrombi enriched with VWF, or both. *P* values calculated using 1-sided Fisher exact test.

d Histologic findings in COVID-19 lung tissue, the myocardium, and pulmonary draining lymph nodes compared to respective controls utilizing staining for platelets (CD42b).

e Histologic findings in COVID-19 lung tissue, the myocardium, and pulmonary draining lymph nodes compared to respective controls utilizing staining for fibrin.

### 3.2 VWF-rich thrombi exclusively found in the lungs of patients with COVID-19

COVID-19 lung tissue samples were collected from autopsies of 28 patients (comprising the cohort presented by Menter et al. [18], with additional 7 cases) who died at a median of 14 days after symptom onset and were compared with samples from a control cohort of patients without COVID-19 (n = 24). The causes of death were COVID-19–associated respiratory failure (n = 27) and myocardial infarction (n = 1). The cause of death in the control group included respiratory failure (n = 19), influenza (n = 5), bacterial pneumonia (n = 5), DAD of other etiology (n = 9), and cardiac death, mainly myocardial infarction (n = 5). There were no significant differences with respect to age, sex, BMI, blood group, or anticoagulant use between both groups. Comorbidities were similarly well balanced, with the exception that hypertension was more prevalent in the COVID-19 group (80% vs 48%; *P* = .03). Hospitalization at the ICU was more common in controls (39% vs 70%; *P* = .03), as was steroid therapy (for the treatment of non–COVID-19 respiratory failure/acute respiratory distress syndrome (ARDS) [12]; 46% vs 8%; *P* = .003), while the rate of mechanical ventilation was similar in both groups (30%, Supplementary Table S2).

Histologically, DAD was more prevalent in the COVID-19 group (100% vs 52%; *P* < .001), explained by the fact that the control collective consisted of not only patients with non–COVID-19 DAD but also patients with cardiac death. The most frequently observed DAD stage in the COVID-19 group was exudative (50%), followed by combined DAD with exudative and proliferative features (32%) and proliferative DAD in the remaining cases (18%) (Table 2).

**Figure 1:**
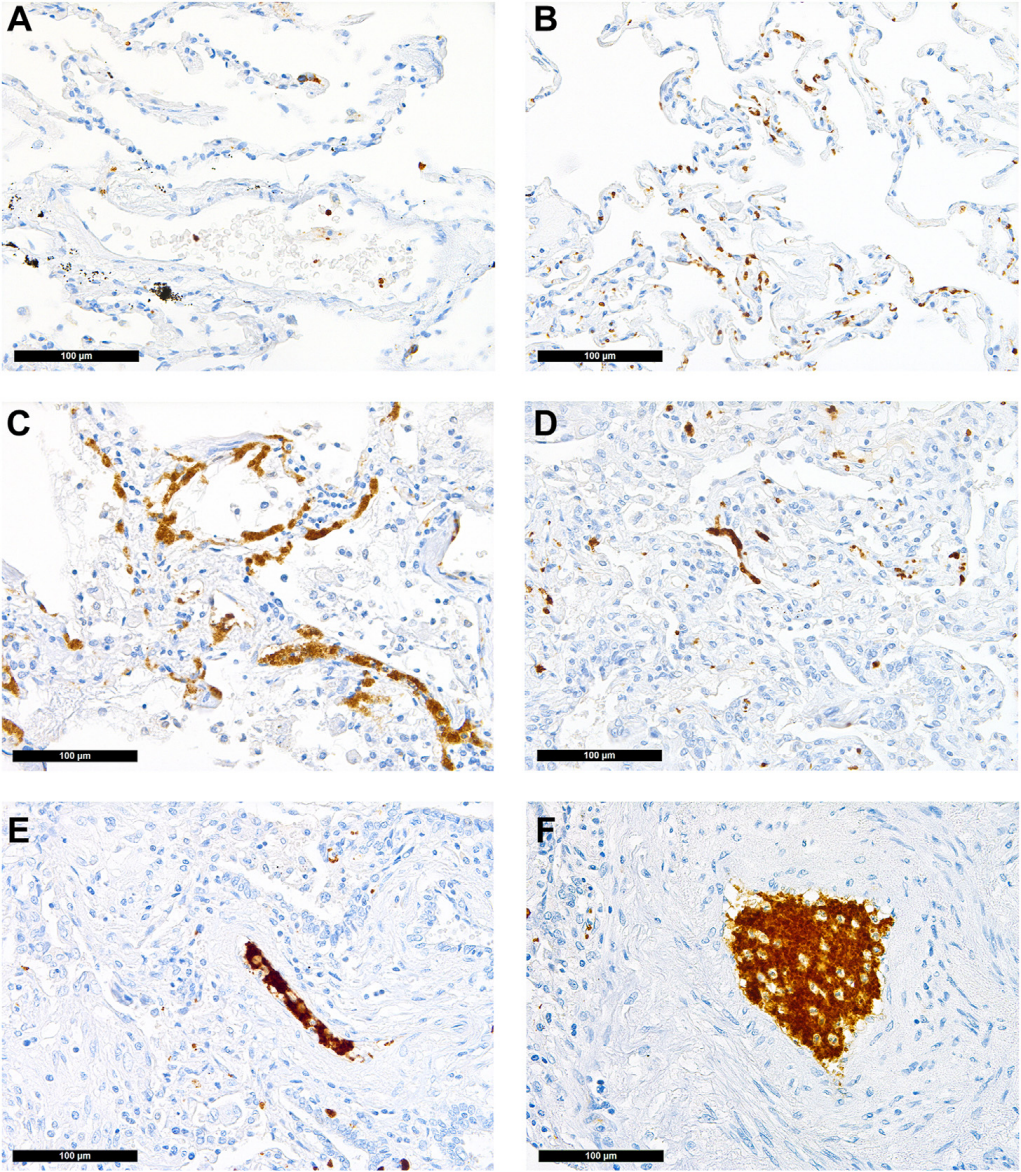
Distribution of platelets in the lung (CD42b immunoperoxidase staining, 400×). (A) Physiologic pattern in control lung tissue: single platelets confined to/within vessels. COVID-19: (B) Increased presence of platelets within alveolar capillaries; (C) CD42b antigenic material/destroyed extravasated platelets within the hyaline membranes of exudative diffuse alveolar damage; (D) platelet thrombus casting a small vessel; (E) platelet thrombus with a few intermingled inflammatory cells in a medium-sized vessel, suggestive of NETosis thrombus; (F) large platelet-enriched NETosis thrombus abundantly intermingled with inflammatory cells.

**Figure 2:**
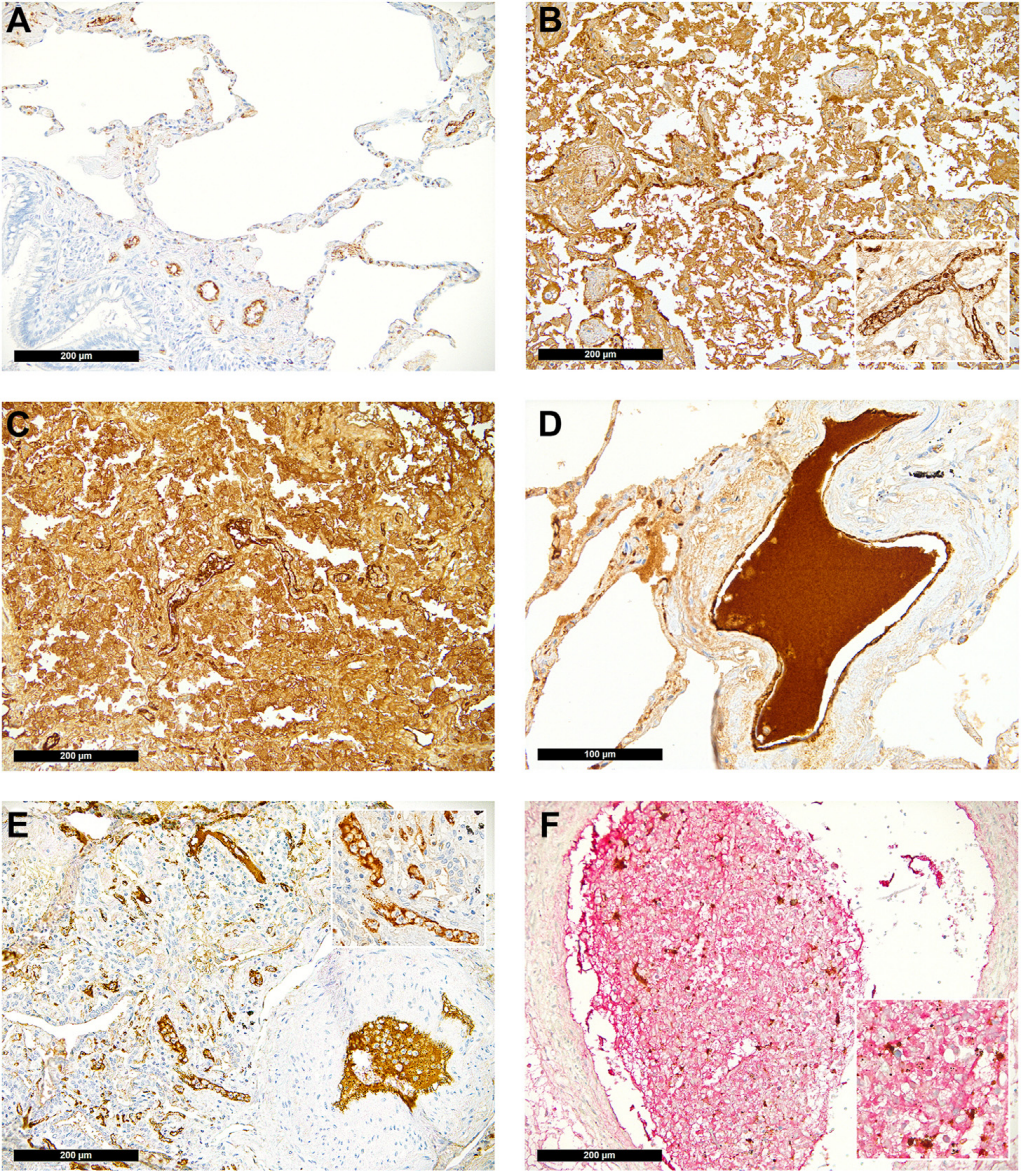
Distribution of von Willebrand factor (VWF) in the lung (immunoperoxidase staining, 200×; inset and D, 400×). (A) Physiologic pattern in control lung tissue with positivity confined to the endothelial lining. (B) VWF in non–COVID-19 diffuse alveolar damage seen outside of the endothelium in the hyaline membranes and desquamated mononuclear cells as well as accentuated within vascular endothelial cells (insert). COVID-19: (C) COVID-19 diffuse alveolar damage with massive presence of VWF in the hyaline membranes, desquamated mononuclear cells, and a NETosis thrombus in the middle of the slide; (D) large VWF^+^ thrombus in a medium-sized vessel; (E) NETosis thrombi in small (also insert) and medium-sized pulmonary vessels enriched for VWF; (F) double staining for VWF and CD42b showing a large NETosis thrombus rich for VWF (red) with entrapped platelets (brown).

Immunohistochemical staining for platelets (CD42b) revealed a physiologic pattern of their distribution confined to the vessels in the majority of the controls but only in one-third of the patients with COVID-19 (63% vs 31%, respectively; *P* = .028; Figure 1A); in 4 COVID-19 cases (14%) and 2 control cases (8%), single scattered megakaryocyte equivalents were detectable (*P* = .674). In 5 COVID-19 cases, platelets were found within the hyaline membranes (Figure 1C), which were not observable in non–COVID-19 DAD (*P* = .038). Compact platelet-rich microthrombi were more frequently observable in patients who died of COVID-19 (36% vs 8%; *P* = .02, Table 2). This difference was more pronounced when the lung tissue samples were analyzed for VWF: in normal lung tissues, VWF is physiologically present in vascular endothelial cells (Figure 2A), and a completely normal pattern of VWF was rare in both groups. However, in patients with COVID-19, we saw less cases with normal pattern of VWF distribution (normal pattern of VWF in patients with COVID-19 vs that in controls: 7% vs 25%, respectively; *P* = .081, Table 2). In patients with pulmonary damage of non–COVID-19 causes, VWF staining was as detectable in desquamated cells as it was in COVID-19 lungs (Figure 2B–D).

Accentuated capillary VWF staining was not exclusive to COVID-19 samples but was found in 29% of the controls. Accentuated capillary staining without thrombi was found in only 11% of COVID-19 cases since samples were graded either as accentuated capillary staining or having microthrombi, and patients with COVID-19 more often had the latter. Importantly, VWF-rich thrombi were exclusively found in patients with COVID-19 (39% vs 0%; *P* < .01; Figure 2D–F), which is a key finding of this study. Similarly, while NETosis was not exclusive to COVID-19 samples, as described earlier in a subgroup of this cohort [52], characteristic NETosis thrombi enriched with VWF were exclusively found in patients with COVID-19 (25% vs 0%; *P* < .01; Figure 2F–G and Supplementary Fig. S1).

**Figure 3:**
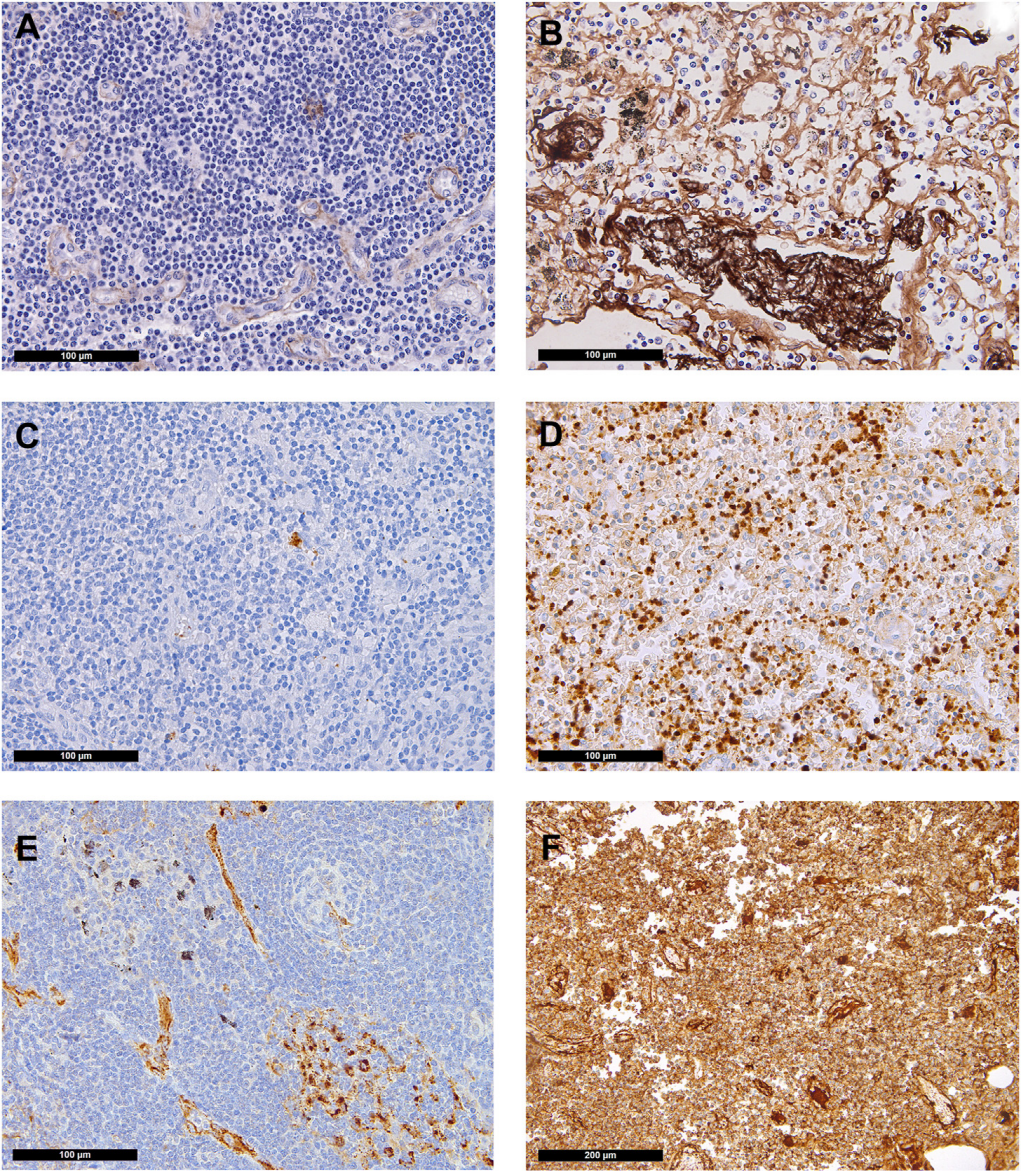
Distribution of fibrin, platelets, and von Willebrand factor (VWF) in the lymph nodes (immunoperoxidase staining, 400×; F, 200×). Control: (A) Minimal presence of fibrin confined to endothelial cells and presence of (C) single platelets and (E) VWF confined to endothelial cells and scattered sinus macrophages in control lymph nodes. COVID-19: (B) Fibrin microthrombi within lymph node sinuses of draining pulmonary lymph nodes of patients with COVID-19; note the significant edema in the background; (D) massively increased platelets, often hemophagocyted in histiocytes, and in sinuses; (F) increased presence of VWF in inflammatory (M2) macrophages.

Taken together, 46% of patients had VWF-rich thrombi, NETosis thrombi enriched with VWF, or both, which was not observed in controls (*P* < .001, Table 2). As expected, presence of VWF^+^ microthrombi in small or medium-sized vessels correlated with the presence of platelet-enriched characteristic NETosis thrombi (ρ = 0.497; *P* = .007). Finally, 12 COVID-19 cases displayed fibrin^+^ microthrombi compared with 2 of the control collective (*P* = .005; Figure 3A, B).

SARS-CoV-2 was detected using reverse transcription-PCR in 85% of lung samples in the COVID-19 cohort, and in all but 1 case (91%) in which VWF^+^ microthrombi were found.

### 3.3 Microthrombi more commonly found in (late) virus-negative COVID-19 hearts

We have previously performed a histopathologic analysis of cardiac microvascular dysfunction with regard to capillary dilatation, fibrin deposition, and microhemorrhages of this cohort of 20 patients with COVID-19 [48] (same donors as the lung cohort), which we now complemented with the analysis of microthrombi in heart tissue samples by immunohistochemical staining for VWF. The control cohort comprised autopsy samples of patients without COVID-19 (n = 9) but with similar sequelae (causes of death: ARDS/DAD, n = 4; pulmonary embolisms, n = 3; and bronchopneumonia, n = 2). Both groups did not differ significantly in comorbidities, including hypertension, except that malignancies were more prevalent in the control group (67% vs 29%; *P* = .038, Supplementary Table S2).

When analyzed for VWF, capillary microthrombi were found in 50% of the patients with COVID-19 vs 44% of the controls, which was not significantly different (Table 2). The same was true when patients who had pulmonary embolism, another prothrombogenic state, were excluded (50% [10/20] vs 33% [2/6]). Regarding fibrin staining results, refer to our previous work [48]. Because of the lack of significant differences in the distribution of VWF and fibrin between COVID-19 and non–COVID-19 hearts, heart tissue samples were not further stained for CD42b.

SARS-CoV-2 was detected using reverse transcription-PCR in 45% of the heart samples and was negative in 55% of the COVID-19 cohort. VWF^+^ microthrombi were slightly more frequently found in PCR-negative instances; ie, in 11 PCR-negative cases, 7 (64%) displayed thrombi, whereas in 9 PCR-positive cases, thrombi were found in only 3 instances (33%). This difference was, however, not statistically significant (*P* = .185).

### 3.4 VWF^+^ histiocytes in COVID-19 lymph nodes correlate with poor COVID-19 outcome

Pulmonary draining lymph nodes of lethal COVID-19 were characterized earlier, including gene expression profiling, and showed morphomolecular changes in microvascular dysfunction [49]. Here, we performed additional analyses encompassing VWF and platelet distribution. Twenty-three samples from patients without COVID-19 (see Methods) were compared to 19 draining pulmonary lymph nodes from patients with COVID-19. As shown earlier, moderate-to-extensive capillary stasis and edema were found in nearly all COVID-19 cases but only in a third of the controls (95% vs 36%, respectively; *P* = .015). Fibrin microthrombi within lymph nodes were present in 32% of the patients with COVID-19 but in none of the controls (*P* = .024; Figure 3A). COVID-19 lymph nodes contained more newly formed (CD105^+^) vessels (median, 68/1.33 mm^2^ vs 44/1.33 mm^2^; *P* = .002) [49].

Immunohistochemical staining for CD42b revealed an increased presence of platelets in COVID-19 (56% vs 35% above the cutoff; *P* = .156; Figure 3C, D). Similarly, platelet-enriched characteristic NETosis thrombi were somewhat more frequent (28% vs 17%; *P* = .336), and VWF^+^ microthrombi tended to be more commonly seen in COVID-19 samples than in controls (35% vs 17%, respectively; *P* = .147; Figure 3F, Table 2). However, while trends were seen, these individual findings were not statistically significant.

In COVID-19 samples, mononuclear cells in the paracortical zones of the lymph nodes frequently displayed positive staining for VWF (40% of COVID-19 cases showed >5% VWF^+^ histiocytes vs 21% of controls; *P* = .038; Figure 3F). This positivity correlated with the time from SOTD, wherein patients with >5% VWF^+^ histiocytes died after a median of 9 days (range, 5.1-12.9 days), whereas patients who were negative died later (median, 19 days; range, 11.3-26.7 days; *P* < .001). Mononuclear VWF^+^ cells were identified as most likely being histiocytes/macrophages by coimmunostaining for transcription factor PU1 and VWF (Supplementary Fig. S2).

SARS-CoV-2 was detected using reverse transcription-PCR in 63% of COVID-19 lymph nodes, similar to and possibly due to their proximity to the lungs, which were positive in 85% of the cases. Four out of 5 cases with NETosis thrombi and 4 out of 7 cases with VWF^+^ thrombi were found in PCR-positive lymph nodes.

## 4 Discussion

While several studies have consistently shown that plasma VWF levels are extremely high and result in an elevated VWF/ADAMTS13 ratio in severe COVID-19, to the best of our knowledge, this is the first study to systematically assess VWF in tissue samples from patients who died of COVID-19 in comparison to well-matched controls.

We found *in situ* evidence of platelet- and VWF-rich thrombi in COVID-19 lungs; higher prevalence of characteristic NETosis thrombi with platelet and VWF enrichment in COVID-19 lungs; and overexpression of *VWF* in pulmonary draining lymph nodes and higher nodal presence of VWF^+^ histiocytes in COVID-19, which correlates with early death [49].

Microthrombotic disease is characteristic but not exclusive to COVID-19 and can also be observed in DAD of other etiologies [16,53]. One meta-analysis comparing 171 patients with COVID-19 and 287 patients with H1N1 influenza reported pulmonary microthrombi in 57% of COVID-19 cases but only in 24% of H1N1 cases [53], while a direct comparison between COVID-19 and H1N1 showed 8 times more microthrombi in COVID-19 lungs [16]. However, most studies assessed microthrombosis by either conventional histochemical stains (hematoxylin and eosin, periodic acid-Schiff, Elastica-van Gieson, or acid fuchsin orange G) [17,47,[54], [55], [56], [57]] or, more rarely, fibrin stain [18,20,56]. In our study, when assessed for CD42b (platelets), pulmonary thrombi were seen in 36% of patients with COVID-19 and only 8% of the controls. Furthermore, when assessed for VWF, accentuated capillary, ie, endothelial, VWF staining was not exclusive to COVID-19. However, VWF^+^ thrombi were detectable in 39% of the patients with COVID-19 but, in contrast to fibrin thrombi, in none of the controls. VWF was also associated with platelet-enriched characteristic NETosis thrombi, which were also more prevalent in COVID-19 samples. More research is certainly needed to determine how specific or exclusive these findings are. Nevertheless and in line with our work, a study by D’Agnillo et al. [20], to the best of our knowledge, is the only other analogous study that described similar findings in their autopsy cohort of 18 patients with COVID-19 who were compared with healthy controls. They showed prominent intravascular and parenchymal VWF immunoreactivity and pulmonary thrombi containing VWF that colocalized with neutrophils and platelets, associated with a shorter SOTD time [20]. Collectively, in the lungs, these observations suggest key pathophysiologic involvement of VWF in COVID-19–associated thrombus formation.

In the myocardium, VWF^+^ microthrombi were found in 50% of the patients with COVID-19 but also in 44% of the controls. This may be due to a small sample size effect of the control group, where half of the patients with microthrombi also had pulmonary embolisms in the setting of a malignant disease, but even after excluding these patients, the similarity remained. Interestingly and possibly explaining these observations, afterload stress due to vessel obstruction has been shown to promote VWF release in cardiac vessels outside the setting of inflammation [58]. While not statistically significant in this study, microthrombi tended to be more often found in COVID-19 myocardium negative for SARS-CoV-2 using real-time reverse transcription-PCR, supporting the hypothesis that cardiac pathology is rather a secondary systemic effect that develops as a result of the hypercoagulable/hyperinflammatory state observed in later, subacute (“virus-cleared”) stages of COVID-19 [48,59] rather than due to direct viral damage. Interestingly, scanning electron microscopy studies found altered microvasculature, an increase in intussusceptive angiogenesis, and ultrastructurally detectable thrombi in COVID-19 hearts [60], findings unapparent in conventional morphology. Hence, microthrombi in our COVID-19 group could also be underrepresented.

Within pulmonary draining lymph nodes, VWF thrombi seemed more commonly observable in the patients with COVID-19 than in the controls, and the overall presence of VWF within lymph nodes was very high, which has similarly been shown on a transcriptional level. In a previous study analyzing the same cohort, *VWF* was among the highest upregulated genes in lymph nodes (fold change, 2.21; *P* = 2.83^−12^ opposed to that in controls) [49]. Whether parallel or secondary to the proposed proangiogenic function of VWF [39], VEGF, which was recently shown to be upregulated by VWF in cancer cells [39], belonged—next to *VWF*—to the highly upregulated genes in lymph nodes of patients with COVID-19 compared to that in controls (fold change, 2.04; *P* = 4.14^−5^). In lung tissue, however, although slight upregulation of VWF in response to tissue damage might be conceivable, eg, due to increased shear stress [61] or hypoxia [62,63], at least in early disease when the endothelium is still functional, in our cohort with lethal COVID-19, we did not find evidence of *VWF* overexpression (ie, no significant difference between groups). As morphologic analysis in lymph nodes showed VWF to be mostly present in histiocytes, we speculate that upregulation of *VWF* in lymph nodes reflects the transcriptional profile of predominant M2-polarized macrophages in response to severe inflammation and endothelial damage [49,64], possibly amplified by hypoxia [62,63], whereas in the lungs, VWF is released from severely damaged vascular endothelium. In view of the vast surface area of pulmonary capillaries alone, spanning >120 m^2^ [65], even without transcriptional upregulation, the contribution of released VWF—upon endothelial decay—to plasma VWF is likely to be substantial. However, whether VWF has an effect on angiogenesis in COVID-19 remains speculative.

Due to the retrospective nature of this work, plasmatic levels of VWF were not available, and D-dimer levels were only available for a few patients (n = 9; 32%) since this cohort stems from an early phase of the pandemic when the prognostic evidence of D-dimer in COVID-19 was yet to become established. Of those, however, in accordance with the literature [2,[20], [21], [22], [23],^25^,^66^], all showed significantly elevated levels.

Our COVID-19 cohort stems from the first wave in Switzerland (March-May 2020; SARS-CoV-2 B.1 lineage), with the benefit that the use of specific medications, which could have served as confounders, was limited. Anticoagulant application was very well balanced between groups, and only 4 patients with COVID-19 received tocilizumab, an IL-6 receptor–blocking antibody. Interestingly, despite the small number of patients receiving tocilizumab and considering that IL-6 can stimulate VWF release and inhibit the cleavage of ultralarge VWF by ADAMTS13 [36], no patient who received tocilizumab had VWF^+^ thrombi in the lungs (while 2 did in the heart). Steroid therapy of any dose (usually methylprednisolone [40 mg/d]) was more prevalent in the control group; thus, possible bias cannot be fully excluded as corticosteroids might decrease endothelial activation and thereby reduce VWF release [21]. Nevertheless, both patients with COVID-19 who received steroids also had VWF^+^ thrombi.

Taken together, we bring *in situ* evidence of VWF-rich thrombi in the lungs, draining pulmonary lymph nodes, and heart tissue of patients who died of COVID-19, which we context as likely attributable to COVID-19, supporting the hypothesis that high levels of VWF and dysregulation of the VWF/ADAMTS13 ratio contribute to the incidence of (micro)thrombotic complications influencing COVID-19 morbidity. This is in line with the growing clinical evidence that increased plasma VWF levels correlate with COVID-19 mortality and the recent evidence that additionally imply an instrumental role of VWF in post–COVID-19 complications [13,14]. Our observations indicate that VWF, possibly perpetuated by (local) relative lack of ADAMTS13 activity, might play a critical role in the pathomechanism of COVID-19 being more than a marker of endothelial damage.

More studies are needed, however, to determine how these findings apply to different COVID-19 variants, for which comparative analyses are scarce to date. Omicron, for example, is more prevalent but less severe, and despite pulmonary embolism being less frequent [11,67], 1 study showed that clotting parameters (not including VWF) are still significantly elevated but may be lower than those with COVID-19 caused by earlier virus strains [68].

We suggest including VWF diagnostics into coagulation analyses of patients with COVID-19, along with D-dimer levels and platelet counts, to deduce a more precise clinical prediction score for COVID-19 severity. Furthermore, at least for selected patients, targeting VWF may be an elegant option for more specific therapy, eg, by supplementation of ADAMTS13 or explicitly by caplacizumab, an anti-VWF nanobody.

## Figures and Tables

**Supplementary Figure 2:**