# Supplementary material for: Von Willebrand factor and the thrombophilia of severe COVID-19: in situ evidence from autopsies
Source: Res Pract Thromb Haemost. 2023 May 18;7(4):100182. doi: 10.1016/j.rpth.2023.100182 (PMC10192064; doi:10.1016/j.rpth.2023.100182)
Supplement: Supplementary Material [file mmc1.pdf]

Suppl. Figure 1

Evidence of NETosis in leukocyte- and VWF-rich thrombi of COVID-19 (630x)

Immunostaining for citrullinated histone H3 (dark blue) and DNA counterstain with Feulgen method (purple) of thrombosis in COVID-19 showing increased amounts of leukocytes in the thrombus along with "shadows" of entrapped erythrocytes (arrowhead) and acellular VWF (not shown, but colocalizing on serial sections), along a fine extracellular meshwork of bluish staining for citrullinated histone H3 (in between asterisks) and purple DNA (arrow), corresponding to NETosis thrombus. For methods, we kindly refer to: Obermayer A, Jakob LM, Haslbauer JD, Matter MS, Tzankov A, Stoiber W. Neutrophil Extracellular Traps in Fatal COVID-19-Associated Lung Injury. Dis Markers. 2021 Jul 30;2021:5566826. doi: 10.1155/2021/5566826. PMID: 34367376.

Suppl. Figure 2

Presence of VWF in macrophages of COVID-19 lymph nodes (400x, insert 630x)

(**A**) Double staining for PU1 (brown), a B-cell and histiocyte-specific transcription factor and VWF (red) in a control lymph node highlighting physiologic presence of VWF confined to endothelial cells without co-expression in PU1+ cells. (**B**) Overexpression of VWF in PU1+ mononuclear cells in COVID-19, most likely M2 macrophages; insert showing unequivocal red (VWF) staining in cells expressing PU1.
