# Supplementary material for: Von Willebrand factor and the thrombophilia of severe COVID-19: in situ evidence from autopsies
Source: Res Pract Thromb Haemost. 2023 May 18;7(4):100182. doi: 10.1016/j.rpth.2023.100182 (PMC10192064; doi:10.1016/j.rpth.2023.100182)
Supplement: Supplementary Tables S1-S3 [file mmc2.pdf]

TABLE S1 | Immunohistochemistry Procedures and List of Antibodies

Immunohistochemistry, except for staining for citrullinated histone H3 and DNA counterstain by the Feulgen method that was executed exactly as described (Obermayer et al.), was performed as per ISO15189 accredited standard diagnostic operating procedures of our institute on an automated stainer (Benchmark Ultra from Roche/Ventana Medical Systems, Tucson, AZ, USA). A list of all utilized antibodies, their dilutions and incubation conditions, and the antigen retrieval procedures are listed in Table S1. To verify/falsify VWF expression in lymph node histiocytes, VWF (AEC-based red read-out)/PU1 (DAB-based brown read-out) double stains were applied; these double stains were performed analogously to what was described elsewhere (Sterlacci W, Savic S, Schmid T, Oberaigner W, Auberger J, Fiegl M, Tzankov A. Tissue-sparing application of the newly proposed IASLC/ATS/ERS classification of adenocarcinoma of the lung shows practical diagnostic and prognostic impact. Am J Clin Pathol. 2012 Jun;137(6):946-56. doi: 10.1309/AJCP77KMKJXNMPMS. PMID: 22586054).

TABLE S1 |

**Marker**	**Antibody source & clone or provider catalogue number**	**Retrieval<br>Dilution<br>Incubation**
CD42b	Neomarkers<br>Clone 42C01	CC1 16 min<br>Ready-to-use<br>12 min
Fibrin	Dako A0080<br>Rabbit polyclonal	Pronase 30 min<br>1:100000<br>overnight
F VIII-R (VWF)	Ventana 760-2642<br>Rabbit polyclonal	CC1 24 min<br>Ready-to-use<br>20 min
PU1	Abcam<br>Clone EPR3158Y	CC1 32 min<br>1:800<br>28 min
Citrullinated histone H3	Abcam ab5103<br>Rabbit polyclonal	Citrate buffer (pH 6.7) at 95°C microwave 10 min<br>1:100<br>60 min

TABLE S2 | Comorbidities and Medication

	COVID-19	Control lungs	Control hearts	Control lymph nodes	All controls	P-values of differences COVID-19 vs.			
**A. Comorbidities**	**(n=28)**	**(n=24)**	**(n=9)**	**(n=23)**	**(n=51**)**	**Lungs only**	**Hearts only**	**Lymph nodes**	**All controls**
Hypertension (%)	20 of 25 (80%)	11 of 23 (48%)	4 of 9 (44%)	4 of 19 (21%)	17 of 46 (37%)	**0.034**	0.082	**<0.001**	**0.001**
Coronary artery disease (%)	12 of 26 (46%)	8 of 22 (36%)	2 of 9 (22%)	4 of 19 (21%)	10 of 45 (22%)	0.565	0.249	0.476	0.061
Diabetes mellitus (%)	7 of 24 (29%)	7 of 22 (32%)	1 of 9 (11%)	5 of 19 (26%)	10 of 44 (23%)	1.0	0.633	0.728	0.571
COPD (%)	4 of 25 (16%)	4 of 21 (19%)	1 of 9 (11%)	4 of 19 (21%)	6 of 43 (14%)	1.0	1.0	0.660	1.0
Malignancy (%)	7 of 25 (28%)	8 of 22 (36%)	6 of 9 (67%)	7 of 19 (37%)	19 of 44 (43%)	0.755	**0.038**	0.728	0.302
Smoking<br>Active (%)<br>History of (%)	9 of 15 (60%)<br>0 of 15 (0%)<br>9 of 15 (60%)	7 of 16 (43%)<br>0 of 16 (0%)<br>7 of 16 (43%)	5 of 9 (56%)<br>2 of 9 (22%)<br>3 of 9 (33%)	12 of 16 (75%)<br>5 of 16 (31%)<br>7 of 16 (44%)	20 of 35 (57%)<br>7 of 35 (20%)<br>13 of 35 (37%)	0.479	0.191	0.149	0.119
**B. Medication**									
Anticoagulation<br>therapeutic (%)<br>prophylactic (%)	20 of 24 (83%)<br>7 of 24 (29%)<br>13 of 24 (54%)	18 of 20 (90%)<br>8 of 20 (40%)<br>10 of 20 (50%)	5 of 7 (71%)<br>4 of 7 (57%)<br>1 of 7 (14%)	9 of 14 (64%)<br>2 of 14 (14%)<br>7 of 14 (50%)	27 of 36 (75%)<br>12 of 36 (33%)<br>15 of 36 (42%)	0.673	0.588	0.435	0.534
Anticoagulation BEFORE admission<br>therapeutic (%)<br>prophylactic (%)	6 of 20 (30%)<br>4 of 20 (20%)<br>2 of 20 (10%)	5 of 20 (25%)<br>4 of 20 (20%)<br>1 of 20 (5%)	2 of 7 (29%)<br>2 of 7 (29%)<br>0 of 7 (0%)	1 of 18 (6%)<br>1 of 18 (6%)<br>0 of 18 (0%)	7 of 40 (18%)<br>6 of 40 (15%)<br>1 of 40 (3%)	1.0	1.0	0.102	0.390
Platelet aggregation inhibition (%)	7 of 22 (32%)	3 of 19 (16%)	0 of 7 (0%)	3 of 17 (17%)	4 of 38 (11%)	0.292	0.134	0.688	0.80
Steroids (%)	2 of 26 (8%)	11 of 24 (46%)	5 of 8 (63%)	4 of 18 (22%)	17 of 45 (38%)	**0.003**	**<0.001**	0.658	**0.006**
Tocilizumab (%)	4 of 27 (15%)	n/a	n/a	n/a	n/a	n/a	n/a	n/a	n/a
Hydroxychloroquine (%)	14 of 27 (52%)	n/a	n/a	n/a	n/a	n/a	n/a	n/a	n/a
Other Antivirals<br>Plasma	10 of 27 (37%)<br>1 of 27 (4%)	n/a	n/a	n/a	n/a	n/a	n/a	n/a	n/a

*: Blood group (BG) distribution in the general population (Blood donor registry, Basel, Switzerland): BG 0= 39,5%, BG A= 44,7%, BG B= 11,1%, BG AB= 4,6%

**: Partially overlapping patients (i.e. lung and lymph node specimens from identical patients); therefore, n ≠ the sum of the n of specific organs.

†: Last available values before exitus; no older than 72h. P-values were calculated by two-sided Fisher's exact test.

TABLE S3 | Blood group distribution and Laboratory results

	COVID-19	Control lungs	Control hearts	Control lymph nodes	All controls	P-values of differences COVID-19 vs.			
**Blood groups***	(data on n=21)	(data on n=22)	(data on n=8)	(data on n=17)	(data on n=42)				
A	9 (43%)	10 (46%)	4 (50%)	9 (53%)	21 (50 %)	1.0	0.675	0.502	0.789
B	1 (5%)	2 (9%)	0	1 (6%)	3 (7%)	1.0	1.0	1.0	1.0
AB	1 (5%)	2 (9%)	0	1 (6%)	2 (5%)	1.0	1.0	1.0	1.0
0	10 (48%)	8 (36%)	4 (50%)	6 (35%)	16 (38%)	0.543	1.0	0.720	0.589
**Laboratory results†**	**(n=28)**	**(n=24)**	**(n=9)**	**(n=23)**	**(n=51**)**	**Lungs only**	**Hearts only**	**Lymph nodes**	**All controls**
D-dimer (µg/ml)	Median 3.7 (mean 8.3) range: 0.43-≥20) (data on n=9)	Median 3.37 (mean 6.28) range: 0.42-18.49) (data on n=5)	Median 3.89 (mean 3.89) (range: 2.15-5.64) (data on n=2)	n/a (n=1: 18.49)	Median 3.37 (mean 5.6; range: 0.42-18.49) (data on n=7)	0.639	0.792	0.614	0.710
INR, median (IQR, range)	1.5 (0.6; 1.2-3.7)	1.25 (0.83; 0.9-3.8)	1.2 (1.43; 1.2-3.2)	1.1 (0.25; 0.9-2)	1.2 (0.65; 0.9-3.8)	0.359	0.523	**0.011**	0.077
CRP, mg/dl, median (IQR, range)	172 (168; 13-512)	118 (182; 4.1-610)	56 (221; 8.6-276)	75 (183; 0.4-335)	90 (177; 0.4-610)	0.115	0.079	0.056	**0.018**
Platelets (10^9/l), median (IQR, range)	197 (223; 16-434)	149 (237; 22-523)	124 (323; 19-631)	211 (224; 42-523)	162 (218; 19-631)	0.266	0.576	0.6	0.276
Leucocytes (10^9/l), median (IQR, range)	8.8 (5.69; 1.75-32.35)	10.79 (12.15; 0.55-39.56)	9.37 (12.23; 1.39-26.82)	10.23 (11.88; 4-30.9)	9.63 (9.6; 0.55-39.56)	0.249	0.922	0.318	0.400
Lymphocytes (10^9/l), median (IQR, range)	0.7 (0.59; 0.21-14.9)	0.74 (0.9; 0.04-11.6)	0.56 (0.28; 0.19-0.61)	1.61 (3.72; 0.57-14.96)	0.74 (1.01; 0.04-14.96)	0.812	0.441	**0.036**	0.633
Neutrophilic granulocytes (10^9/l), median (IQR)	7.03 (5.49; 3.2-28.85)	9.45 (13.47; 0.43-28.45)	6.4 (12.3; 0.68-15.7)	6.88 (9.91; 2.19-27.65)	7.82 (8.81; 0.43-28.45)	0.209	0.849	0.922	0.787

*: Blood group (BG) distribution in the general population (Blood donor registry, Basel, Switzerland): BG 0= 39,5%, BG A= 44,7%, BG B= 11,1%, BG AB= 4,6%

**: Partially overlapping patients (i.e. lung and lymph node specimens from identical patients); therefore, n ≠ the sum of the n of specific organs.

†: Last available values before exitus; no older than 72h. P-values were calculated by two-sided tests (i.e. Fisher's exact test and Mann-Whitney U test).
